# Tetramethylpyrazine reverses high-glucose induced hypoxic effects by negatively regulating HIF-1α induced BNIP3 expression to ameliorate H9c2 cardiomyoblast apoptosis

**DOI:** 10.1186/s12986-020-0432-x

**Published:** 2020-01-31

**Authors:** Shih-Ping Liu, Marthandam Asokan Shibu, Fuu-Jen Tsai, Yuan-Man Hsu, Chang-Hai Tsai, Jing-Gung Chung, Jai-Sing Yang, Chih-Hsin Tang, Shulin Wang, Qiaowen Li, Chih-Yang Huang

**Affiliations:** 1 Graduate Institute of Biomedical Sciences, China Medical University, Taichung, Taiwan; 2 College of Medicine, Hualien Tzu Chi Hospital, Buddhist Tzu Chi Medical Foundation, Tzu Chi University, Hualien, Taiwan; 3 School of Chinese Medicine, College of Chinese Medicine, China Medical University, Taichung, 40402 Taiwan; 4 China Medical University Children’s Hospital, China Medical University, Taichung, Taiwan; 5 Department of Biological Science and Technology, China Medical University, Taichung, Taiwan; 6 Department of Pharmacology, School of Medicine, China Medical University, Taichung, Taiwan; 7 Department of Medical Research, China Medical University Hospital, China Medical University, Taichung, Taiwan; 8 Department of Cardiology, The Sixth Affiliated Hospital of Guangzhou Medical University, Qingyuan People’s Hospital, Qingyuan, 511518 Guangdong China; 9 Department of Biotechnology, Asia University, Taichung, Taiwan

**Keywords:** HIF-1, Diabetes mellitus, Food flavoring, Caspase, Hypoxia

## Abstract

**Background:**

Diabetic patients are highly vulnerable to hypoxic injury, which is associated with hypoxia induced BNIP3 expression that subsequently activate apoptosis. Our previous research show that Tetramethylpyrazine (TMP), a food flavoring agent, represses the hypoxia induced BNIP3 expression attenuate myocardial apoptosis. In this study, we evaluate the effect of TMP to provide protection against hypoxia aggravated high-glucose associated cellular apoptosis.

**Methods:**

The cytoprotective effect of TMP against high glucose induced cellular damages was determined on embryo derived H9c2 cardiomyoblast cells that were subjected to 5% hypoxia for 24 h and subjected to different duration of 33 mM high glucose challenge. Further, the involvement of HIF-1α and BNIP3 in cellular damage and the mechanism of protection of TMP were determined by overexpression and silencing HIF-1α and BNIP3 protein expression.

**Results:**

The results show that hypoxic effects on cell viability aggravates with high glucose challenge and this augmentative effect is mediated through BNIP3 in H9c2 cardiomyoblast cells. However, TMP administration effectively reversed the augmented HIF-1α levels and BNIP3 elevation. TMP improved the survival of H9c2 cells and effectively suppressed apoptosis in H9c2 cells. Further comparison on the effects of TMP on H9c2 cells challenged with high glucose and those challenged with hypoxia show that TMP precisely regulated the hypoxic intensified apoptotic effects in high-glucose condition.

**Conclusion:**

The results clearly show that flavoring agent-TMP attenuates cytotoxicity amplified by hypoxia challenge in high glucose condition by destabilizing HIF-1α.

## Background

Coronary heart disease is a critical condition that accounts for about 80% of death among people with diabetes. Diabetic patients are more prone to cardiac events such as stroke as diabetes mellitus is associated with macrovascular manifestations such as atherosclerosis and medial calcification [1, 2]. Numerous studies have revealed hyperglycemia as an independent risk factor that can directly inflict cardiac damage, thereby contributing to diabetic cardiomyopathy [3]. Initial responses of cardiac cells to hyperglycemic condition include abnormal metabolism, abnormal fetal gene expression and defects in cellular organelles [4]. Cardiomyocyte death follows as a consequence to these abnormalities and forms a reason for subsequent collagen accumulation and reduced contractile function [5].

Although, diabetes is an independent risk factor for CVD, evidences show that hyperglycemia, contributes to myocardial damage following ischemic events [6].

Hypoxic conditions are seen to be the general cause of cardiac cell death in pathological conditions including diabetes [7–9]. Previous studies have shown that the probable mechanism of hypoxia-induced apoptosis is through hypoxia induced stabilization and nuclear translocation of hypoxia-inducible factor-1α (HIF-1α) protein. Activation of HIF-1α induces downstream proteins including Bcl-2 adenovirus E1B nineteen-kilodalton interacting protein 3 (BNIP3) and insulin-like growth factor binding protein 3 (IGFBP-3) expression. BNIP3 forms a stable homodimeration complex that is inserted into the mitochondrial membrane causing mitochondrial damage resulting in mitochondria-dependent apoptosis. In addition, IGFBP-3 enhances cellular apoptosis by sequestering the survival factor IGF-1 thereby blocks the IGF1R/PI3K/Akt signaling activation [10–12]. Such events associated with excessive hypoxia have a strong correlation with cardiac diseases.

Under normoxia conditions, the transcription of the *bnip3* gene is repressed by transcription factors NFκB and E2F4 [13]. In addition, *bnip3* is one of the most abundant genes induced by HIF-1 under hypoxic conditions. BNIP3 mRNA and protein levels are enhanced due to a HRE that occurs in the proximal promoter for *bnip3* that binds to the heterodimer of HIF-1, inducing BNIP3 expression [14]. Hypoxia-induced high-level transcription of *bnip3* is mediated by E2F1 releasing from E2F4 and FoxO3 to enhance HIF-1α transcriptional function [15–17]. Our previous studies show that prolonged-hypoxia induced HIF-1α stimulated BNIP3 and enhanced IGFBP-3 activation and their effect on survival pathway and mitochondria-dependent cardiomyocyte apoptosis are similar in neonatal cardiomyocytes and in H9c2 cardiomyoblast cells [18]. H9c2 cell lines show a identical hypertrophic responses as those observed in primary neonatal cardiomyocytes when exposed to hypertrophic factors like angiotensin-II, endothelin-1 [19]. The cell line is also used for cardiotoxicity analyses and to understand stress associated mechanisms and myocyte damage including apoptosis and necrosis [20]. Moreover, H9c2 cells are well known to mimic primary cardiomyocytes in their responses to hypoxia and in addition, they are more similar to primary cardiomyocytes in their energy metabolism patterns. Therefore, H9c2 cells are more appropriate to simulate cardiac ischemic effects [21].

Previous reports show that, Tetramethylpyrazine (TMP) found in food products such as potato fries, fermented cocoa bean, tea, coffee, bread, beer, spirits, peanuts and various dairy-foods is a potential food ingredient to provide cardio-protection. Our previous reports elucidated the rescue effect of TMP on H9c2 cells against hypoxia-induced apoptosis that involves suppression of HIF-1α, BNIP3 and IGFBP3 [22–24]. In the present study, we further explore the beneficial effects of TMP induced suppression BNIP3 on hypoxia aggravated cardiac apoptosis in hyperglycemic condition.

## Materials and methods

### Cell culture and treatment

H9c2 cardiomyoblasts from the American Type Culture Collection (ATCC,CRL-1446) (Rockville, MD, USA) were cultured in 100-mm or 60-mm culture dishes in Dulbecco’s modified Eagles medium (DMEM, Sigma-Aldrich, USA) supplemented with 100 μg/mL penicillin (Gibco, Waltham, MA, USA), 100 μg/mL streptomycin (Gibco), 2 mM glutamine (Gibco), and 10% Clontech fetal bovine serum (Hyclone, GE Healthcare Life Sciences, Pittsburgh, PA, USA) inside humidified air (5% CO_2_) at 37 °C. The control cells were exposed to media containing 5.5 mM D-glucose and for high-glucose challenge the cells were exposed to 33 mM D-glucose according to previous reports [4, 7, 25].

### Examination of protein expressions by Western blotting

A total of 5 × 10^5^ cells of H9c2 cells were plated onto 10 cm dish and incubated at 37 °C for treated with high glucose for 12 h and then combined in the hypoxia environment and for another 24 h. To isolate total proteins, H9c2 cells were washed with cold PBS and resuspended in lysis buffer (50 mM Tris, pH 7.5, 0.5 M NaCl, 1.0 mM EDTA, pH 7.5, 10% glycerol, 1 mM BME, 1% IGEPAL-630 and a proteinase inhibitor cocktail (Roche Molecular Biochemicals, Germany). After incubation for 30 min on ice, the supernatant was collected by centrifugation at 12000 g for 15 min at 4 °C. The protein concentration was determined using the Bradford method (Bio-rad, USA). Samples containing equal amounts of protein (50 μg) were loaded and analyzed using Western blot analysis. Briefly, proteins were separated by 10% SDS-PAGE and transferred onto PVDF membrane (Millipore, Belford, Massachusetts, USA). Membranes were blocked with blocking buffer (5% non-fat dry milk, 20 mM Tris-HCl, pH 7.6, 150 mM NaCl, and 0.1% Tween 20) (Sigma-Aldrich, St. Louis, MO, USA) for at least 1 h at room temperature. Membranes were incubated with primary antibodies (caspase-3, HIF-1α, IGFBP3, Akt, Bcl-2, Bak, Bax and β-actin (Santa Cruz Biotechnology, Inc. Santa Cruz, California, USA), BNIP3, cleaved caspase-9, cleaved caspase-3, phosphorylated-Akt Ser473 (p-Akt) (Cell Signaling, Danvers, MA, USA), in the above solution on an orbit shaker at 4 °C overnight. Following primary antibody incubations, membranes were incubated with horseradish peroxidase-linked secondary antibodies (anti-rabbit, anti-mouse, or anti-goat IgG) (Santa Cruz).

### TUNEL assay

The TUNEL (TdT-mediated digoxigenin- dUTP nick-end labeling) method was carried out with a commercially available in situ apoptosis detection kit (Roche Molecular Biochemicals). H9c2 were stained according to the manufacturer’s protocol. TUNEL-positive cells were identified with a fluorescent microscope using an excitation wavelength in the 450–500 nm range and a detection wavelength in the 515–565 nm range (green). The percentage of apoptotic cells was calculated by dividing TUNEL-positive cells by the total number of cells visualized in the same field. Three digitized images with similar total cell numbers were selected from each cover slip for counting and averaging and were considered as one independent experiment. Three independent experiments were then averaged and statistically analyzed.

### siRNA transfection

Double-stranded siRNA sequences targeting HIF-1α and BNIP3 mRNAs were obtained from Sigma-Aldrich (St. Louis, Missouri, USA). The non-specific siRNA (scramble) was non-targeting. Cells were cultured in 60-mm well plates in medium. siRNA transfection was carried out with transfection reagent (PureFection™, System Biosciences, Mountain View, CA.) following the manufacturer’s protocol. Specific silencing was confirmed by immune blotting with cellular extracts after transfection.

### Gene overexpression through transient transfection

Cells with 50% confluence were placed into fresh culture medium containing serum 2 h before the transient transfection. pCDNA3-HAyun-FoxO3a [26] plasmids were then transfected into the cells for 24 h using the PureFection™ Nanotechnology-based Transfection Reagent (System Biosciences, CA, USA) following the manufacturer’s protocol. In each experiment, the gene overexpression efficiency was measured by western blot (data not shown).

## Results

### High glucose aggravates hypoxia induced BNIP3 gene expression in H9c2 cardiomyoblast cells

**Fig. 1:**
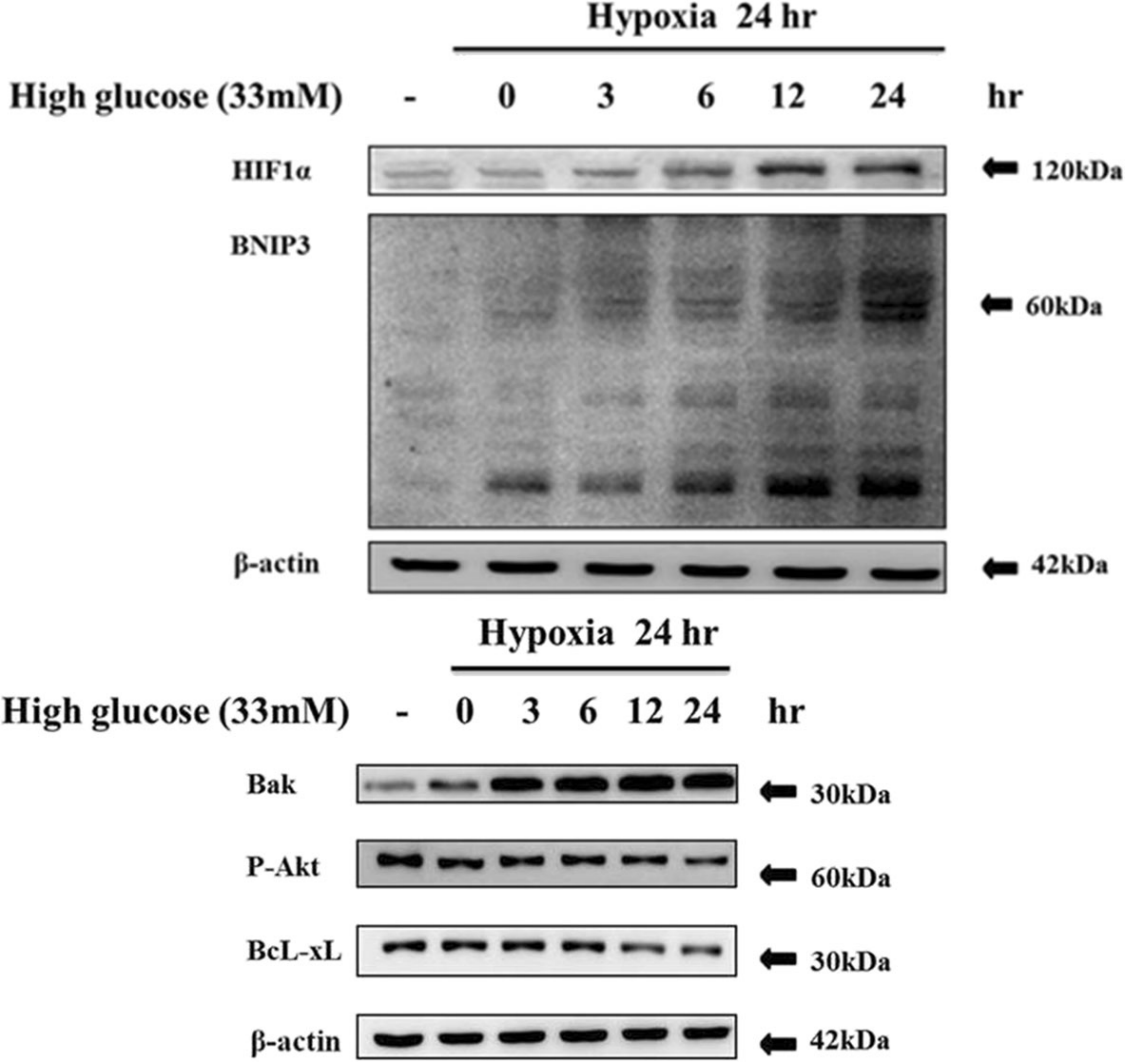
Effects of high-glucose and hypoxia on BNIP3 expression in H9c2 cells. Cells were cultured in high-glucose medium (33 mM) and incubated in hypoxia (5%) for different time-periods as indicated. Wetern blotting analysis show increase in hypoxia associated protein expression such as HIF1α and BNIP3 that was correlated with increase in pro-apoptotic protein Bak and decrease in anti-apoptotic proteins P-Akt and BcL-xLl(*n* = 3).

H9c2 cells under 33 mM high glucose when exposed to hypoxic conditions for 24 h showed aggravated increase in BNIP3 expression in a time dependent manner (Fig. 1). Increase in BNIP3 expression was also correlated with increase in pro-apoptotic proteins such as Bak and decrease in cell survival associated anti-apoptotic proteins such as p-Akt and BcL-xL.

### High glucose stress under hypoxia enhances HIF-1α nuclear translocation and induces BNIP3 expression

**Fig. 2:**
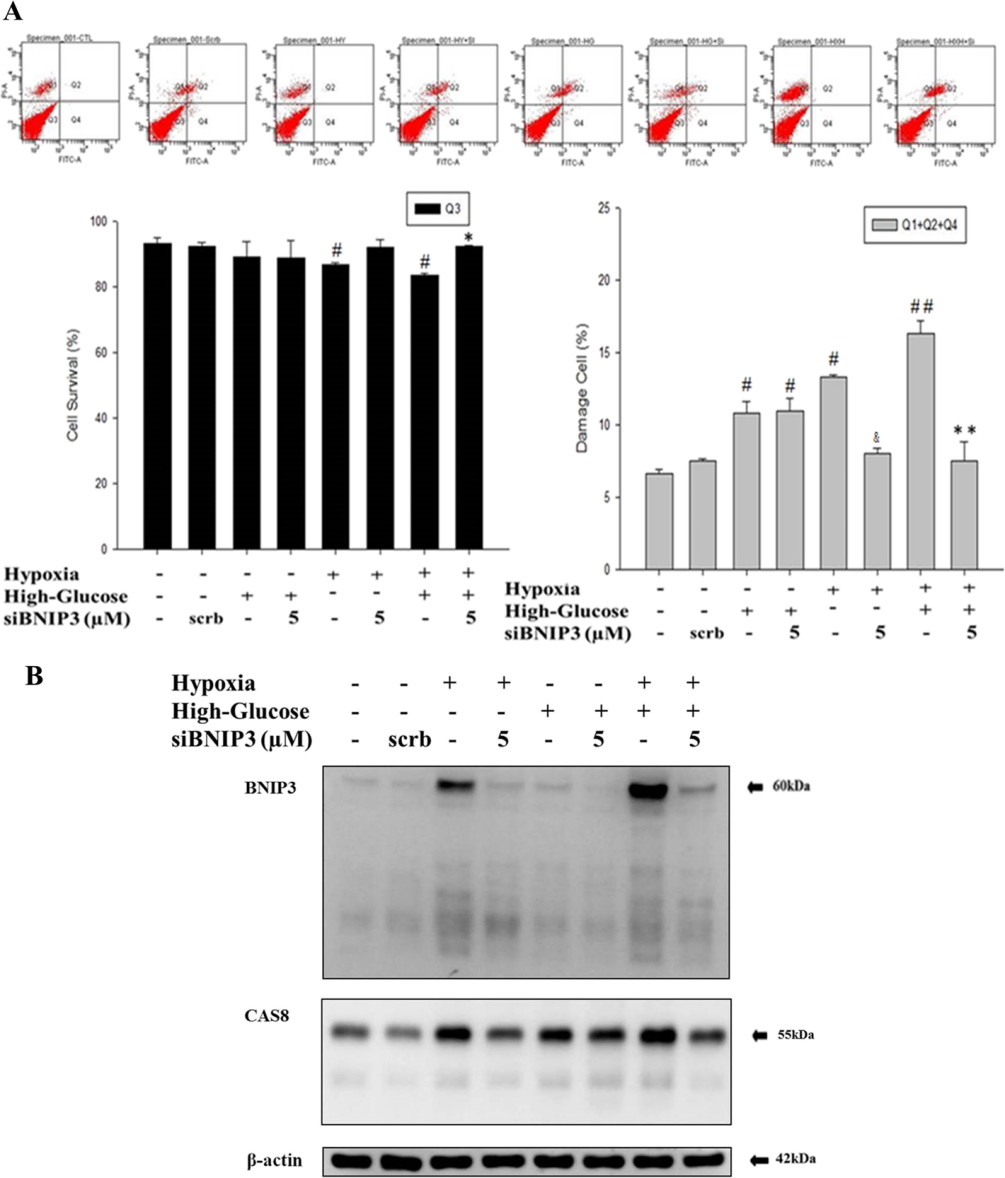
Hypoxia aggravates High-glucose induced cellular apoptosis via BNIP3 regulation. **a** Flow cytometry analysis show aggravated cell death in H9c2 cells up on exposure to hypoxia under high-glucose challenge. The results also show reversal of hypoxia-high glucose induced cell death in the presence of siBNIP3 (*n* = 3). The cells without labeling represents living (Q3) cells; the cells labeled with Annexin V-FITC (Q2, Q4) or propidium iodide (Q1, Q2) were respectively cells in apoptosis and in necrosis (**b**) Western blotting analysis show the correlation of BNIP3 expression with caspase 8 activation (*n* = 3) up on hypoxia exposure in high glucose condition

To ensure whether high glucose can aggravate hypoxia induced cardiomyoblast apoptosis we exposed H9c2 cells to high glucose conditions and hypoxia and analyzed by flow cytometry. The results show that the synergistic effect of hypoxia and high glucose stress elevated the apoptotic cell death (Fig. 2a) which also correlation with BNIP3 levels (Fig. 2b). Further, hypoxia induced-Bnip3 was significant inhibited when BNIP3 was silenced by siRNA transfection and the reduction in BNIP3 levels were correlated with reduction in apoptosis (Fig. 2).

### TMP inhibits cellular apoptosis triggered by high glucose stress under hypoxia and downregulates BNIP3 levels

**Fig. 3:**
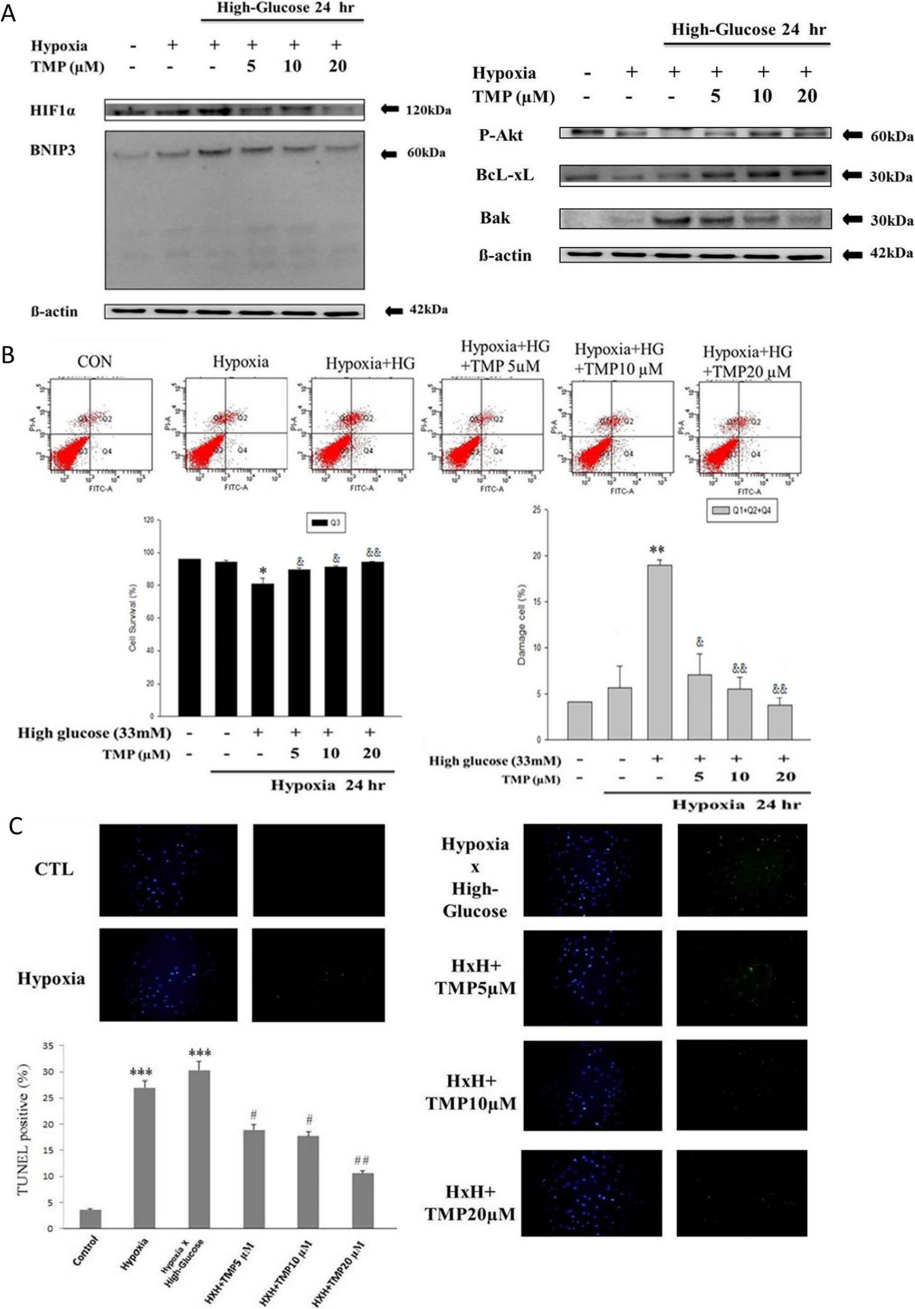
Effect of Tetramethylpyrazine on elevated HIF-1α and the downstream BNIP3 in H9c2 cells under hypoxia and high glucose condition. **a** Western blotting analysis (*n* = 3) show increase in both HIF1α and BNIP3 levels under hypoxia in high-glucose conditions. **b** Flow cytometry analysis (*n* = 3) show TMP induced modulation on the effect of hypoxia in H9c2 cells under high glucose condition; cells without labeling represents living (Q3) cells; the cells labeled with Annexin V-FITC (Q2, Q4) or propidium iodide (Q1, Q2) were respectively cells in apoptosis and in necrosis. **c** Microphotographs (400X magnification) of TUNEL assay show the ameliorating effect of TMP on cell death induced by hypoxia under high-glucose condition (*n* = 3)

To investigate the effect of TMP on H9c2 cells under synergistic hypoxia and high glucose stress 5 μM, 10 μM and 20 μM of TMP was treated to the cells cultured in high glucose media under hypoxia. The Western blotting results show that treatment with TMP dose-dependently suppressed the levels of HIF-1α and BNIP3. Reduction in the levels of HIF-1α and BNIP3 was also correlated with increase in the level of p-Akt and BcL-xl (Fig. 3a). Flow cytometric analysis also show that treatment with TMP reduces in the cellular apoptosis induced by the synergistic effect of high glucose and hypoxia (Fig. 3b). The TUNEL assay also shows that TMP reduces the number of apoptotic nuclei in H9c2 cell challenged with both hypoxia and high glucose (Fig. 3c).

### TMP attenuates the catastrophic apoptotic events induced by high glucose under hypoxia regulating the synergistic increase BNIP3 expression in H9c2 cells

**Fig. 4:**
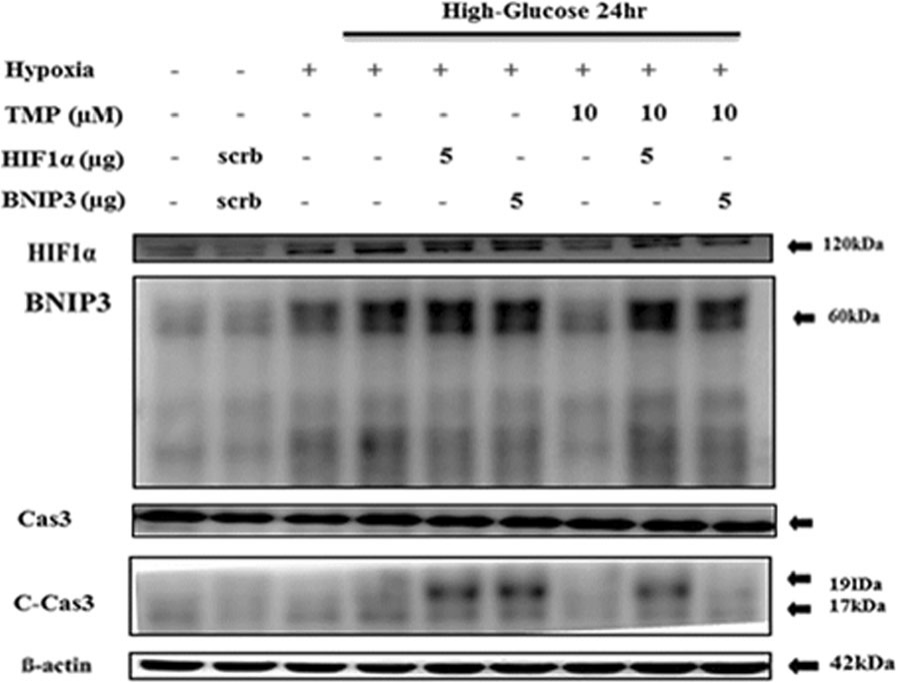
TMP attenuates hypoxia and high-glucose induced BNIP3 expression and apoptosis. Western blotting analysis (*n* = 3) show TMP induced modulation in HIF1α and BNIP3 levels in H9c2 cells under hypoxia and in hypoxia in combination with high-glucose and in cell overexpressing either HIF1α or BNIP3

We further over expressed HIF-1α and BNIP3, incubated with high glucose and hypoxia, and treat with 10 μM of TMP. We found that both HIF-1α and BNIP3 increased during hypoxia but the expression of apoptotic markers were considerably increased when co-challenged with both high glucose and hypoxia. Further increase in BNIP3 and HIF-1α by overexpression also showed elevated caspase activation. However, treatment with TMP was more effective in regulating BNIP3 associated caspase activation (Fig. 4).

In summary, our results show that hypoxia exposure under high-glucose challenge triggers aggravated cellular apoptosis via elevation of BNIP3 levels and TMP administration effectively inhibited high-glucose aggravated hypoxic cell death via down regulation of BNIP3 expression.

## Discussion

Chronic hypoxia in the presence of high glucose leads to progressive acidosis of cardiomyocytes in culture. We previously demonstrated HIF-1α as an important transcription factor in ischemic embryo rat heart [27, 28] [29], and increased protein level of HIF-1α is associated with high glucose treatment. Another study indicated that hypoxia/acidosis genes such as BNIP3, IGFBP-3, hemeoxygenase 1 (HO-1), pyruvate dehydrogenase kinase 1 (PDK1), prolyl hydroxylases (PHDs) and HIF-2 were upregulated during ischemia [30]. This study evaluates the involvement of HIF-1α and BNIP3 in hypoxic condition under high glucose stress.

A critical response to hypoxia in cells and tissues is mediated through changes in the transcriptional induction of a series of genes that participate in angiogenesis, iron metabolism, glucose metabolism, and cell proliferation/survival that are regulated by the hypoxia-inducible factors (HIFs) [31, 32]. In this study, we found that hypoxia induced BNIP3 expression is enhanced by high glucose in a time dependent manner in H9c2 cardiomyoblast cells. Further, HIF-1α expression was found to be directly proportional to BNIP3 levels in hypoxic condition which was further elevated in high-glucose condition. Moreover, apoptosis in H9c2 cardiomyoblast cells also increased with increasing BNIP3 levels observed in hypoxic condition.

Chronic hyperglycemia is the major cause to many diabetic complications. Generally, cells regulate the glucose transport during hyperglycemia in order to maintain cellular metabolic homeostasis. Disturbances in the glucose homeostasis in diabetic condition is associated with deterioration in the glucose transport. BNIP3 is a BH3 protein that promotes mitochondrial destruction and triggers mitochondrial death by increasing the mitochondrial membrane permeability and subsequent release of cytochrome *c.* Our data suggest that BNIP3 expression is significantly upregulated following 24 h of hypoxic stress in H9C2 cardiomyoblast under high glucose condition.

Previous reports showed that HIF-1α induced by hypoxia-acidosis could target the promoter of BNIP3 to transcribe these genes and further induce programmed cell death. Here, we further evaluated if elevated HIF-1α mediate BNIP3 to enhance cardiomyocyte apoptosis. BNIP3 has been reported to be a potent inducer of autophagy in adult rat cardiac myocytes. The present results show that BNIP3 expression is augmented by high glucose under hypoxic condition and resulted in mitochondria-dependent apoptosis in cardiomyocytes. These findings clearly indicate that HIF-1α-induced BNIP3 signaling is an important modulator of hypoxia-induced mitochondrial apoptosis. A previous study showed that BNIP3 activity is induced by hypoxia in an HIF1-dependent manner [33–36].

Our previous results show that food ingredients and phytochemicals exhibit various diverse biological activities [37–41]. Particularly various phytoextracts and their active principles have shown to provide effective cardioprotection against various challenges [42–50]. Cocoa consumption has been previously linked to cardiac health and various mechanisms have been proposed for the cardioprotective effects of cocoa [51]. TMP is one of the major bioactive component of cocoa liquors and in Chinese black vinegar [52, 53]. Moreover, *Ligusticum chuanxiong Hort*, a commonly used traditional Chinese medicine used for empiric treatment of cerebrovascular and cardiovascular diseases for many centuries also contains TMP as its major bioactive principle. This traditional Chinese medicines has been widely used in clinical treatments for vascular disease, and it has been efficient in animal stroke models and is known to preserve the structural and functional integrity of mitochondria [22]. Our previous study has shown that *Ligusticum chuanxiong* extracts inhibits the inflammatory markers p-NFkB and increases the survival marker p-Akt expression. TMP, also known as ligustrazine is known to possess a wide range of biological activities including antioxidant, anti-inflammatory, antitumor, properties and provide cyto-protection to kidneys, brain and heart [23, 54–57]. TMP is also known to improve microcirculation and can be potentially used to treat thrombosis as they attenuate platelet aggregation [58, 59].

Our results reveal that TMP inhibits cell apoptosis of H9c2 cells in high-glucose condition with hypoxia, a condition that imitate those in diabetes patients during ischemic injury. We further observed that HIF-1α and BNIP3 induced cell apoptosis in H9C2 cardiomyoblast cells increases during hypoxia and is further fortified in hypoxia with high-glucose stress, however the effects of BNIP3 was significantly inhibited by TMP treatment.

## Conclusions

In this study, by using over expression of HIF-1α and BNIP3 and with TMP treatment, we concluded that high glucose associated hypoxia induced myocardial cell apoptosis mediated by HIF-1α dependent signaling and the activation of BNIP3 is attenuated with TMP treatment.

## Data Availability

The datasets used and/or analysed during the current study are available from the corresponding author on reasonable request.
